# Human liver and pancreas innervation: resolving 3D neurohistological challenges and advancing insights

**DOI:** 10.1186/s12929-025-01194-y

**Published:** 2025-11-10

**Authors:** Chih-Yuan Lee, Fu-Ting Hsiao, Chien-Chia Chen, Shiue-Cheng Tang

**Affiliations:** 1 Department of Surgery, National Taiwan University Hospital, Taipei, Taiwan; 2 Institute of Biotechnology, National Tsing Hua University, Hsinchu, Taiwan; 3 Department of Medical Science, National Tsing Hua University, Hsinchu, Taiwan

**Keywords:** 3D histology, Autofluorescence, Autolysis, Autonomic nervous system, Glucose homeostasis, Human liver, Human pancreas, High-refractive-index polymer, Intrapancreatic ganglia, Liver innervation, Metabolic regulation, Pancreas innervation, Photobleaching, Super-resolution imaging, Tissue clearing

## Abstract

The human liver and pancreas are central to metabolic regulation, with the autonomic nervous system orchestrating processes that maintain glucose homeostasis and respond to dynamic physiological demands—ranging from acute energy mobilization during stress to postprandial glucose uptake and storage. However, visualizing and examining the intricate three-dimensional (3D) neural networks within clinical liver and pancreas specimens remains challenging, as conventional two-dimensional (2D) histological methods cannot fully resolve the spatial complexity of autonomic innervation in the liver and pancreas. This review identifies and discusses key biological and technical factors—including tissue autofluorescence, autolysis, photobleaching, and steatosis—that compromise the reliability of 3D neurohistological analysis of the human liver and pancreas. We also highlight emerging optical and chemical methodologies that enable high- and super-resolution 3D tissue imaging, improving signal fidelity, preserving structural detail, and supporting consistent, reproducible analysis. Ultimately, these advances aim to facilitate precise mapping of human liver and pancreas innervation, offering deeper insight into the neural regulation of nutrient assimilation, glucose utilization, and metabolic homeostasis in both physiological and pathological contexts.

**Supplementary Information:**

The online version contains supplementary material available at 10.1186/s12929-025-01194-y.

## Background

### A dynamic partnership in glycemic regulation

The liver and pancreas are the primary metabolic organs essential for maintaining glucose homeostasis, with their functions tightly regulated by the autonomic nervous system [1–5]. Physiologically, the endocrine pancreas controls blood glucose levels through the opposing actions of its key islet hormones, insulin and glucagon. The two hormones are delivered to hepatocytes via the portal venous system, linking pancreatic hormone secretion to hepatic glucose regulation. Insulin promotes hepatic glycogenesis, while glucagon stimulates glycogenolysis, together ensuring balanced glucose levels in response to hormonal and neural cues. For instance, marked hypoglycemia (blood glucose < 40 mg/dl) has been shown to activate the sympathetic nervous system, prompting glucagon release from pancreatic α-cells [6]. This, in turn, stimulates hepatic glycogenolysis, effectively raising blood glucose levels to restore normoglycemia. In contrast, patients with unstable type 1 diabetes may exhibit impaired autonomic function [7], disrupting both pancreatic glucagon secretion and hepatic glycogenolysis. Such disruptions can lead to severe hypoglycemia during insulin therapy—a life-threatening complication in diabetes management [8].

Other physiological states that engage the autonomic nervous system include the “fight-or-flight” and “rest-and-digest” responses, predominantly mediated by the sympathetic and parasympathetic branches, respectively [9]. During acute stress, sympathetic activation triggers catecholamine release from the adrenal medulla, enhancing hepatic glucose production to meet emergency energy demands [10]. Conversely, in the postprandial state, parasympathetic activation and gut hormones stimulate insulin secretion from pancreatic β-cells [11, 12], promoting hepatic glycogenesis and lowering blood glucose levels. These intricate neuroendocrine interactions between the nervous system, pancreas, and liver underscore the necessity for detailed anatomical characterization to elucidate hepatic and pancreatic innervation patterns in both health and disease. Such investigations are crucial for advancing our understanding of metabolic regulation and the coordinated control of the two organs. Clinically, a more precise understanding of this innervation could inform new strategies for neuromodulatory therapies aimed at restoring metabolic balance in diabetes, obesity, and fatty liver disease.

### The essential role of clinical specimens in translational liver and pancreas research

Experimental animal models, particularly rodents, have been instrumental in elucidating the molecular mechanisms and cellular complexities of hepatic and pancreatic physiology, as well as their associated pathophysiology. However, fundamental differences in lifespan, nutritional requirements, and metabolic profiles between rodents and humans result in notable variations in tissue architecture, vascularization, and innervation. These species-specific differences must be carefully considered when translating experimental findings into clinically relevant contexts.

**Fig. 1:**
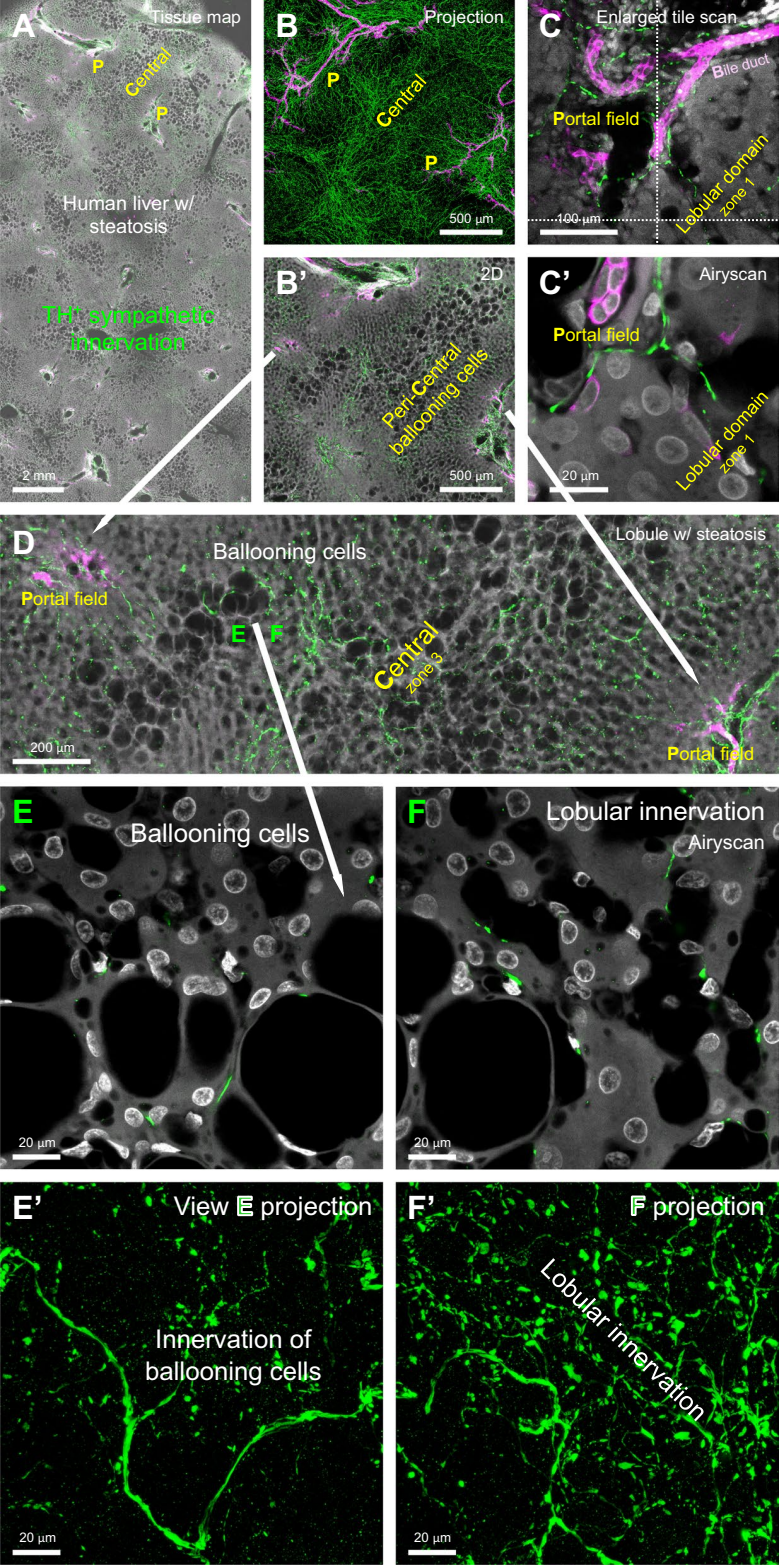
Human liver with steatosis: panoramic-to-Airyscan super-resolution neurohistology. **A** Steatotic lobules obtained from the distal resection margin of hepatocellular carcinoma (male, 55 years old, right lobe). Sympathetic nerves are labeled with tyrosine hydroxylase (TH, green). The central and portal (P) regions at the top are magnified in panels **B** (projection) and **C** (2D image). Bile ducts are labeled with CK19 (magenta), nuclei with DAPI (white), and steatotic areas are visualized using transmitted light signals (gray). **B, B’** Projection and 2D images showing lobular innervation and peri-central ballooning hepatocytes. **C, C’** Sympathetic nerves and bile ducts in the portal field entering the lobule. **D** Enlarged view of the central field highlighting sympathetic innervation of ballooning hepatocytes. **E, F** 3D Airyscan imaging further magnifies the lobular microenvironment of ballooning hepatocytes. Sympathetic nerves in these areas are presented in **E’** and **F’** (projection views). Curved sympathetic nerve fibers closely follow the contours of ballooning cells in **E’**, suggesting direct contact and potential modulation. Note that the enlarged volumes of hepatocytes in **E** and **E’** exhibit a local reduction in nerve density (compared with **F** and **F’**). *Supplementary Video 1 provides a depth-resolved example of 3D Airyscan super-resolution imaging of the steatotic microenvironment*

For instance, studies on hepatic innervation have highlighted substantial interspecies variability in neural distribution [13–16]. Unlike humans, rodents lack intra-lobular sympathetic nerves in the liver—a structural difference that limits their ability to accurately model certain human-specific neural and pathological features. In human non-alcoholic fatty liver disease (NAFLD), for example, ballooning hepatocytes within the intra-lobular domain are directly exposed to sympathetic axonal contacts (Fig. 1 and **Supplementary Video 1**), a morphological feature that rodent models fail to replicate adequately.

**Fig. 2:**
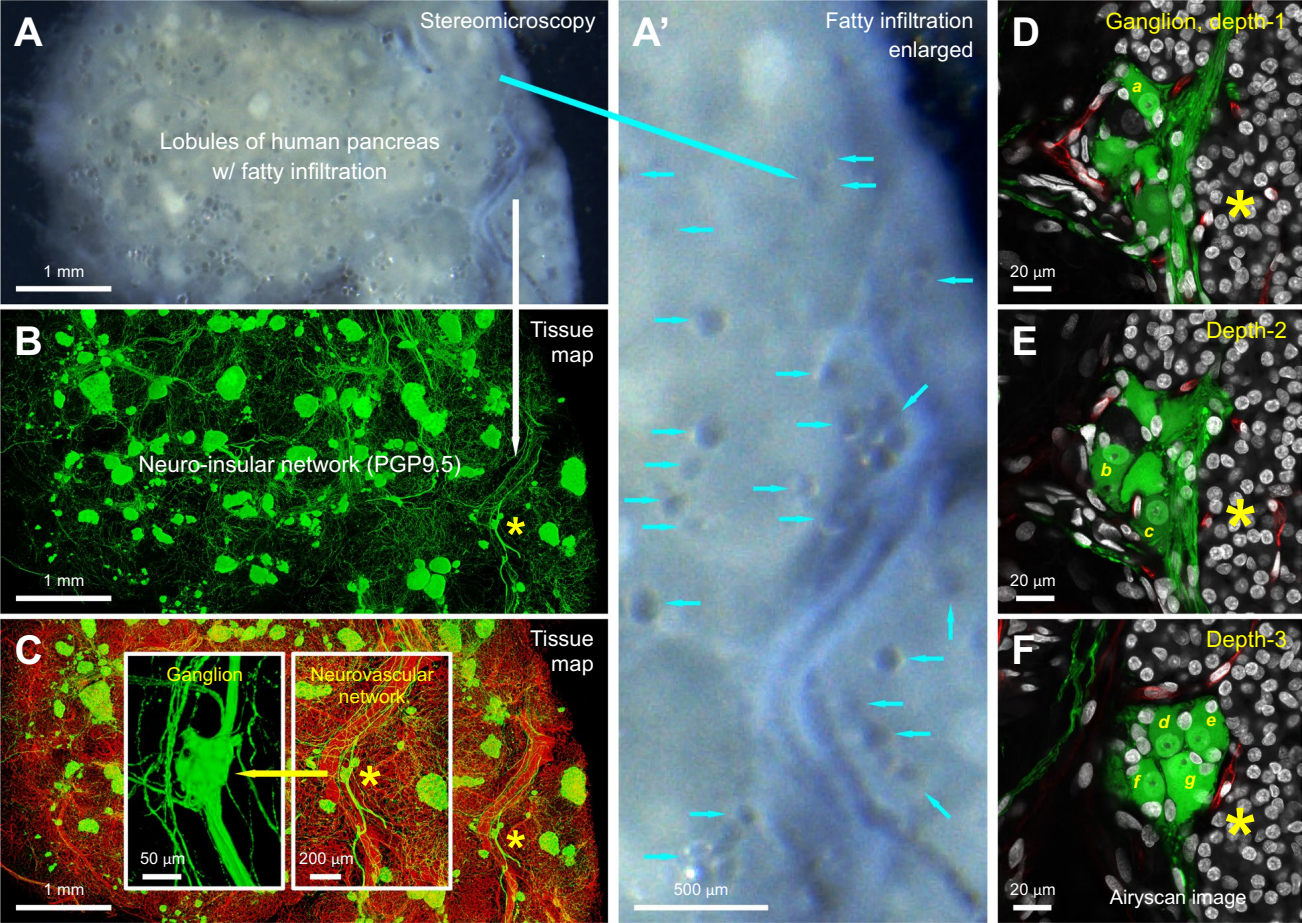
Human pancreas with steatosis (fatty infiltration): panoramic-to-Airyscan super-resolution neurohistology. **A, A’** Vibratome section of a steatotic donor pancreas (male, 43 years old, tail region). Arrows in **A’** indicate infiltrated adipocytes located in both peri-lobular and intra-lobular spaces. **B, C** Projections of neuro-insular and neurovascular networks. **A–C** examine the same macroenvironment. Green: neuroendocrine marker PGP9.5; red: endothelial marker CD31. PGP9.5 staining highlights both nerves and islets. Asterisks in **B** and **C** indicate a PGP9.5⁺ ganglion (enlarged in the insets), further magnified in **D–F**. **D–F** In-depth Airyscan imaging of neurons within the ganglion. Depth-resolved images identify seven neurons: “a” in **D**, “b” and “c” in **E**, and “d–g” in **F**. PGP9.5⁺ neuronal somas are shown, each containing a nucleus with a distinct nucleolus. Green: PGP9.5; red: CD31; white: DAPI. *Supplementary Fig. 1 provides a depth-resolved example of a ganglion labeled with the glial marker S100B in the human pancreas with steatosis*

Similarly, differences in pancreatic fat deposition underscore the limitations of rodent models. Pancreatic steatosis—a condition characterized by fat infiltration into pancreatic tissue—is commonly observed in adult humans, and its severity is closely associated with metabolic disorders such as obesity and type 2 diabetes (Fig. 2 and **Supplementary Fig. 1**) [17–20]. Although both humans and mice exhibit peri-lobular fat accumulation in pancreatic steatosis, intra-lobular fatty infiltration is rare in mice. This distinction is critical, as intra-lobular adipocytes in humans appear to remodel and integrate with the pancreatic parenchyma. Consequently, studies on pancreatic innervation in the contexts of obesity, diabetes, and pancreatic neoplasia must account for this remodeled microenvironment; ignoring the role of adipocytes may lead to an oversimplified understanding of disease pathology.

Given the necessity of using human specimens in translational research to avoid misleading results, the following sections examine the challenges and strategies for applying 3D high- and super-resolution neurohistology to the analysis of human liver and pancreas tissues. The goal is to apply 3D imaging techniques capable of resolving structures across multiple scales—from macroscopic (centimeter-scale) views to sub-micrometer precision—enabling accurate mapping and characterization of innervation while minimizing artifacts that could generate false-positive or false-negative results.

### False-positive signals: tissue autofluorescence

Autofluorescence presents a major challenge in fluorescence-based immunohistochemistry, particularly when visualizing innervation patterns in human liver tissues. The liver contains abundant endogenous fluorophores—including nicotinamide adenine dinucleotide (NADH) and flavin adenine dinucleotide (FAD), both key components of cellular respiration and metabolic pathways [21–23]; bilirubin, a bile pigment [24]; lipofuscin, an aging pigment derived from lipid peroxidation [25, 26]; and porphyrins, which participate in heme synthesis [27]. All of these emit fluorescence across a broad spectrum. Their emissions frequently overlap with commonly used fluorescent dyes, complicating signal interpretation, generating false positives, and obscuring specific labeling in fluorescence imaging.

NADH emits blue fluorescence (excitation: 320–380 nm, emission: 420–460 nm), which can interfere with signals from DAPI, a commonly used nuclear stain. Similarly, the green-yellow fluorescence of FAD (excitation: 450–500 nm; emission: 520–550 nm) and bilirubin (excitation: 460–490 nm; emission: 520–530 nm) overlaps with fluorophores such as Alexa Fluor 488, which is commonly used for green fluorescence labeling. Additionally, lipofuscin (excitation: 360–450 nm, emission: 500–700 nm) and porphyrins (excitation: ~ 400 nm, emission: 600–700 nm) emit red fluorescence, which can interfere with dyes like Alexa Fluor 594 and Texas Red. This spectral overlap complicates the identification of neuronal markers, especially in liver cells affected by oxidative stress or aging, where lipofuscin and porphyrins accumulate. The resulting background fluorescence lowers the signal-to-noise ratio, making it challenging to accurately visualize nerve fibers, varicosities, and vesicles in fluorescence-based 3D immunohistochemistry.

Beyond endogenous fluorophores, blood-derived components also contribute to autofluorescence in liver biopsy specimens due to the liver’s extensive vascularization [28, 29]. Residual blood components exhibit strong intrinsic fluorescence across the visible spectrum, further interfering with imaging. Similarly, pancreatic biopsies are affected by blood-derived autofluorescence [30], which persists even in donor pancreases that have been perfused before dissection [31]. These artifacts vary across clinical specimens, necessitating careful interpretation—particularly when dark regions appear in transmitted light microscopy of optically cleared 3D specimens [31, 32].

### Strategies for autofluorescence reduction

Several chemical and optical methods have been developed to suppress autofluorescence in fixed specimens. Chemical quenchers—such as TrueBlack and Sudan Black B [33–36]—are applied post-staining to improve visualization of specific labeling targets. TrueBlack reduces lipofuscin autofluorescence, while Sudan Black B is beneficial for specimens with high lipid content. However, careful optimization is essential because excessive use of quenchers can also reduce the intensity of desired fluorescent signals, leading to false-negative results.

Chemical bleaching offers an additional strategy for suppressing autofluorescence in fixed tissues [37–39]. Bleaching agents such as hydrogen peroxide and ammonium chloride react with endogenous fluorophores by breaking down their molecular structures through oxidative processes. These agents are often applied as pre-staining treatments to remove background fluorescence. Although chemical bleaching is cost-effective and simple to implement, excessive exposure can lead to false-negative results due to tissue and antigen damage from free radical generation. These radicals may modify or destroy specific epitopes, thereby hindering the accurate detection of molecular targets.

**Fig. 3:**
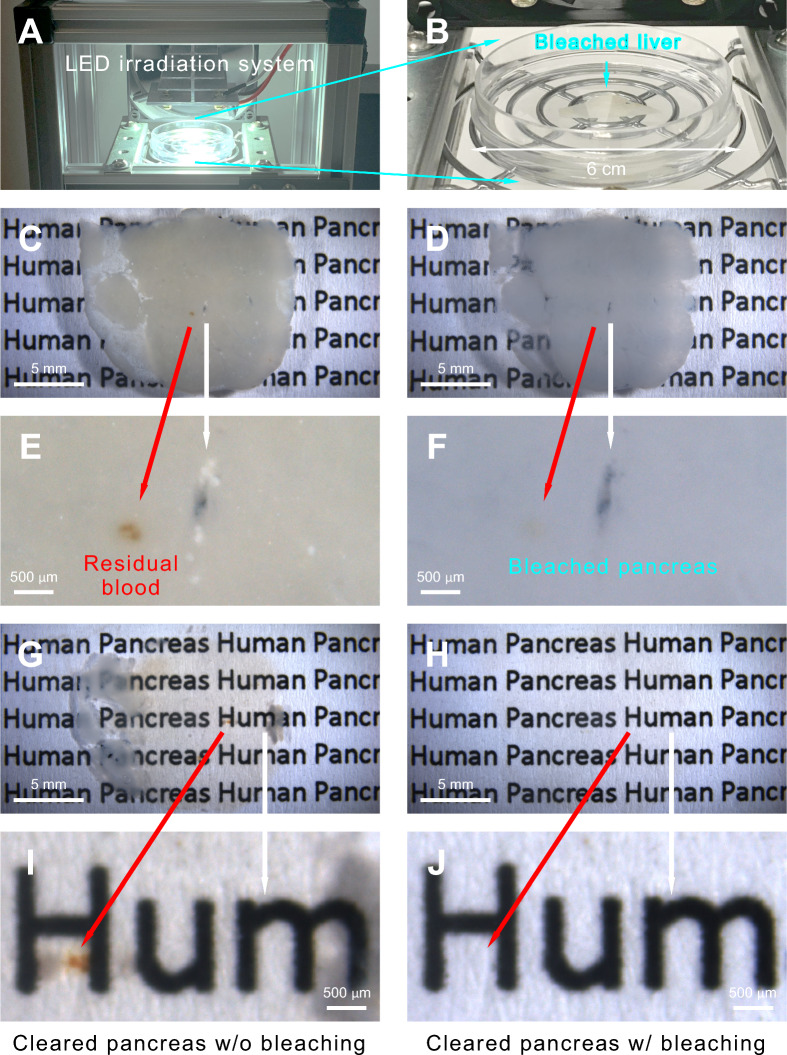
Reduction of pigments in human liver and pancreas specimens using LED-based photochemical bleaching. **A, B** LED irradiation system using white light for decolorization of human liver specimens immersed in 0.3% hydrogen peroxide. **C–F** Comparison of a human pancreas vibratome section (350 µm thickness) before **C, E** and after photochemical bleaching **D, F**. Arrows indicate magnified areas. Photochemical bleaching effectively removed residual blood, which was clearly visible before treatment (compare **E** vs. **F**). **G–J** Integration of tissue clearing with LED-based decolorization in pancreas specimen preparation. Comparison of cleared tissue without and with LED-based decolorization (**G, I** vs. **H, J**). Arrows indicate magnified areas. The combined bleaching and tissue clearing processes effectively removed residual blood (**I** vs. **J**), improving photon penetration and minimizing imaging artifacts in 3D high- and super-resolution microscopy

An emerging approach for reducing tissue autofluorescence is the integration of light-emitting diode (LED) irradiation with chemical bleaching—known as photochemical bleaching—as a combined pre-staining treatment [40–42]. This approach enhances tissue decolorization by effectively degrading natural fluorophores and pigments through a photon-initiated free-radical bleaching reaction. High-intensity LED light can be tuned to specific wavelengths or applied as broad-spectrum radiation (e.g., white light; Fig. 3A, B) to reduce background fluorescence. Figure 3C–J compare untreated and treated human pancreas specimens before and after photochemical bleaching. This method can also be integrated with tissue clearing—a critical step in rendering specimens transparent using high-refractive-index media for 3D immunohistochemistry (see [43–45] for reviews)—as shown in Fig. 3H, J. In human liver and pancreas neurohistology, LED-based bleaching effectively reduces background autofluorescence in the visible light spectrum, resulting in high-definition, depth-resolved images (Figs. 1 and 2).

### False-negative results: tissue autolysis

The pancreas is highly susceptible to postmortem autolysis due to its abundant digestive enzymes. Autolytic changes in acinar cells can begin within an hour after death, with the rate and severity influenced by factors such as temperature, cause of death, clinical conditions, and—most critically—the postmortem interval [46–48]. To preserve morphological features and antigen integrity for neurohistological analysis, immediate tissue fixation after surgical resection is essential. Ideally, specimens should be fixed in formaldehyde at 4 °C to halt enzymatic degradation. Although methanol-based fixatives like CytoLyt have been used in cell blocks and surgical pathology, their application in fine-needle aspiration samples of the pancreas has yielded suboptimal results [49]. In CytoLyt-fixed cases, investigators have found that pancreatic or tumor autolysis has compromised morphological evaluation, underscoring the impact of digestive enzyme activity on histological outcomes even under non-physiological conditions.

To mitigate autolysis, protease inhibitors such as aprotinin and chymostatin have been incorporated into tissue culture media to extend the viability of human pancreatic slices in culture conditions [50, 51]. This approach can also be adapted for washing surgical or needle biopsy specimens—both to remove residual blood (thereby reducing autofluorescence in 3D imaging, as discussed previously) and to minimize enzymatic activity prior to formalin fixation, preserving tissue architecture and antigenicity. While tissue autolysis is a well-recognized cause of false-negative results in histological analysis, partially degraded nucleic acids, proteins, and membrane components dispersed throughout the tissue may also produce false-positive signals—such as autofluorescence [52]—interfering with 3D labeling in high- and super-resolution neural network imaging. Therefore, forensic pathology criteria for assessing pancreatic autolysis [53], using gold-standard H&E histology, should be consistently applied in specimen quality control to prevent erroneous outcomes in pancreatic neural tissue analysis.

In liver neurohistology, autolysis is also a concern, though its effects are generally less pronounced than in the pancreas [53]. Nonetheless, the same preventive measures—prompt fixation, low-temperature preservation, protease inhibitors, and rapid tissue processing—are critical for ensuring high-quality liver specimens. These precautions are particularly important in liver innervation studies, where tissue integrity is essential for accurate visualization and interpretation.

### False-negative results: photobleaching in high-power fluorescence neurohistology

Photobleaching is a major limitation in fluorescence microscopy, wherein fluorophores lose their ability to emit light due to photochemical degradation from prolonged light exposure. This issue is particularly significant in high-power and super-resolution imaging, where the extended use of high-intensity lasers and high-power objectives increases the likelihood of photobleaching [54–57]. As a result, critical innervation patterns in the liver and pancreas may be misinterpreted or lost, leading to false-negative findings in both normal and pathological conditions.

**Fig. 4:**
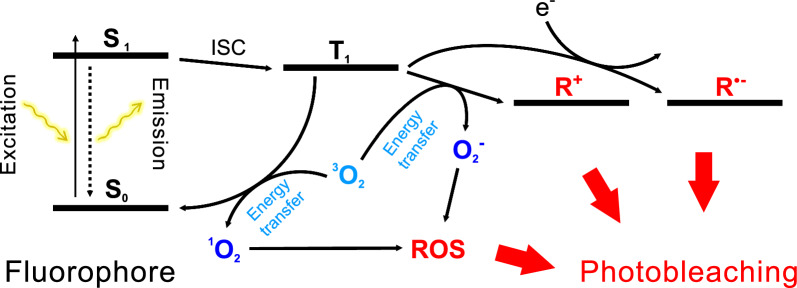
Schematic illustration of molecular oxygen (^3^O₂) involvement in fluorophore photobleaching. The diagram depicts the role of molecular oxygen (^3^O₂) in the photochemical degradation of fluorophores. Key transitions and intermediates in the photobleaching process include: S₀: fluorophore in the ground state; S₁: fluorophore in the first singlet excited state; ISC: intersystem crossing; T₁: the first triplet excited state; ROS: reactive oxygen species; R⁺: radical cation; R•⁻: radical anion. These interactions contribute to irreversible fluorophore degradation, ultimately reducing fluorescence signal intensity

In tissue clearing reagents—where specimens remain in a liquid environment—fluorophores are particularly susceptible to photobleaching due to interactions with reactive molecules, primarily oxygen [54–56]. Photon absorption triggers photochemical reactions that generate free radical species, leading to fluorophore degradation and reduced stability over time (Fig. 4). Notably, the same oxidative processes responsible for photobleaching are also leveraged to reduce autofluorescence by degrading endogenous pigments and fluorophores, as shown in Fig. 3. However, in fluorescence microscopy, photobleaching constitutes a significant challenge, as it restricts observation time and reduces quantitative imaging accuracy.

Beyond its impact on overall imaging accuracy, photobleaching poses an even greater challenge in 3D super-resolution fluorescence neurohistology, where several factors further exacerbate its effects. First, prolonged imaging increases fluorophore exposure to high-intensity light, accelerating signal degradation. Second, small neuronal structures—such as nerve fibers, varicosities, and synaptic vesicles—contain only sparse distributions of fluorophores, making each signal crucial for accurate visualization. Additionally, focused laser beams further intensify photobleaching, particularly in methods like stimulated emission depletion (STED) microscopy [58], where depletion lasers force fluorophores into non-fluorescent states in highly localized regions. Given these constraints, mitigating photobleaching is essential to ensure reliable imaging of neural structures in both healthy and diseased tissues.

## Strategies to minimize photobleaching

### Oxygen removal and antioxidant scavengers

One strategy to mitigate photobleaching is to reduce oxygen in the imaging environment, as oxygen drives photochemical reactions that convert fluorophores into reactive oxygen species (ROS), accelerating their degradation (Fig. 4). To counteract this process, enzymatic oxygen scavengers (e.g., glucose oxidase/catalase systems) and antioxidants (e.g., ascorbic acid) are often included in imaging reagents, though their effectiveness in extending fluorophore stability remains inconsistent. [59, 60]. While these agents help neutralize ROS and slow the rate of photobleaching, they also introduce technical challenges. Some antioxidants and scavengers exhibit intrinsic fluorescence or coloration, interfering with imaging signals and reducing data accuracy. Alternatively, imaging in an anoxic environment can further reduce oxygen-related degradation, but maintaining a completely oxygen-free environment is technically demanding and may lead to variability in imaging parameters and system reproducibility.

### Light sheet fluorescence microscopy

Light sheet fluorescence microscopy offers an alternative method to reduce photobleaching by minimizing light exposure and phototoxicity [61–63]. This technique uses a thin sheet of light to illuminate only the imaging plane, drastically reducing fluorophore excitation and overall photobleaching. Light sheet microscopy is particularly useful for imaging large structures, such as nerve trunks and bundles of the liver [64, 65], enabling rapid and comprehensive data collection while preserving fluorescence. However, while light sheet microscopy excels in preserving fluorescence, it is not designed for the high-power magnifications required to resolve subcellular structures such as individual nerve fibers, varicosities, or synaptic vesicles. Consequently, this approach is less suitable for applications requiring super-resolution imaging of fine neuronal structures.

### Tissue solidification with antifade reagents

Another strategy to prevent photobleaching is embedding fluorophores in solid or semi-solid polymer-based antifade reagents, such as ProLong Gold or VECTASHIELD [66–69]. These compounds reduce molecular diffusion and limit exposure to oxygen, thereby decreasing ROS generation and extending fluorophore stability.

While effective in 2D imaging, polymer-based antifade reagents pose challenges in 3D imaging. Their high molecular weight and viscous nature hinder penetration into thick tissues, resulting in uneven embedding. This creates optical heterogeneity along the imaging path, exacerbating refractive index mismatches and increasing light scattering, which degrades image quality in deeper regions of the specimen.

**Fig. 5:**
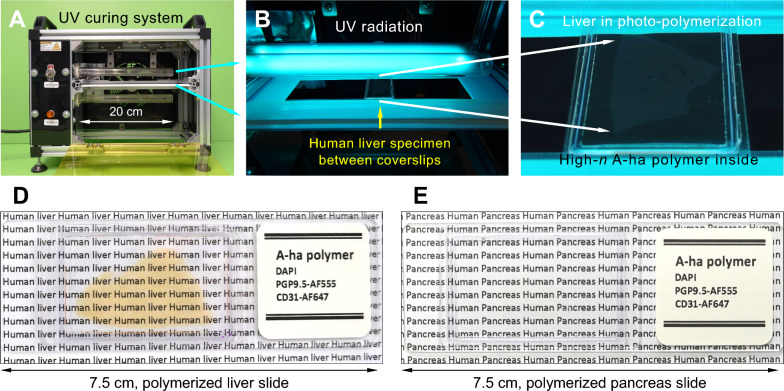
UV-induced photo-polymerization for preparing transparent human liver and pancreas specimens in solid A-ha polymer for antifade fluorescence microscopy. **A–C** UV radiation for photo-polymerization. A 30-min irradiation was performed using two pairs of UV lamps (emission peak at 253.7 nm) positioned above and below the specimen. A 350-µm-thick liver specimen, placed between two coverslips using an iSpacer (SunJin Lab), was immersed in a monomer solution containing acrylamide and N-hydroxymethyl acrylamide (86.7% monomer mass fraction; molar ratio 1:1) with the photoinitiator Irgacure 2959 (0.04% mass fraction). This setup enabled photo-polymerization between the coverslips, leading to the synthesis of the high-refractive-index (high-*n*) A-ha polymer, which renders the specimen transparent. **D, E** Transparent human liver and pancreas embedded in A-ha polymer on a standard microscope slide. The polymerized specimens with labeling of nuclei (DAPI), nerves (PGP9.5), and blood vessels (CD31) are antifade, allowing for repeated 3D and super-resolution imaging, as well as sample transfer across laboratories, making them highly valuable for translational research [70]. *Examples of high-definition videos derived from the polymerized human liver and pancreas specimens are presented in Supplementary Videos 1–3*

A novel approach to mitigating photobleaching and light scattering in 3D neurohistology uses the chemical and optical properties of high-refractive-index polymers to integrate solid-state tissue clearing and antifade fluorescence imaging into a unified process. By utilizing small acrylamide-based monomers for effective tissue penetration followed by UV-induced polymerization, Hsiao et al. successfully prepared solid, transparent human pancreas and liver specimens, as well as mouse kidney, liver, and brain samples [70]. The UV-polymerized specimens demonstrated a significant reduction in photobleaching and improved imaging clarity at subcellular resolution. Moreover, the process was optimized for polymerization directly on standard microscope slides (Fig. 5), enabling compatibility with various commercially available systems, including Zeiss Airyscan microscopy [70–72], Leica SP8 with STED [72], Zeiss lattice structured illumination microscopy [73], and Nikon spatial array confocal (NSPARC) microscopy [74]. In clinical tissue analysis, the final preparations of UV-polymerized specimens resemble standard 2D pathological sections (Fig. 5D, E), offering optical clarity, chemical stability, and long-term preservation.

## Steatosis and its impact on 3D liver and pancreas neurohistology

Steatosis, characterized by excessive lipid accumulation within the liver and pancreas, is frequently associated with metabolic disorders such as obesity, insulin resistance, and type 2 diabetes [19, 75]. While hepatic steatosis primarily results from lipid deposition within hepatocytes, pancreatic steatosis is driven by adipocyte infiltration and/or deposition within the pancreatic parenchyma. Despite these differences, both conditions involve structural remodeling that may alter neurovascular networks and impair organ function. In the liver, lipid accumulation displaces hepatocyte nuclei, leading to macrovesicular steatosis and potential disruptions in neurovascular organization within hepatic lobules [76]. Similarly, in the pancreas, fat accumulation occurs around blood vessels, ducts, and interlobular spaces, altering stromal composition and reshaping local neural architecture [19].

The effects of steatosis on organ innervation may be further compounded by associated inflammation and fibrosis. In the liver, inflammatory responses triggered by steatosis can lead to neuropathic alterations—including hypersensitivity or impaired nerve function—that may disrupt the brain–vagus–liver axis [77, 78]. This dysfunction has been linked to metabolic dysregulation, exacerbating insulin resistance and reinforcing fat accumulation in a cyclical manner. In the pancreas, chronic inflammation associated with steatosis may affect the parasympathetic and sympathetic regulation of insulin secretion and exocrine function, potentially contributing to metabolic imbalance. Moreover, in pancreatic intraepithelial neoplasia (PanIN), adipocyte infiltration is often observed alongside stromal changes [32, 79], suggesting a possible link between fat accumulation, neural remodeling, and early-stage neoplastic progression.

Beyond its metabolic consequences, steatosis presents significant technical challenges for 3D neurohistology, particularly in fluorescence imaging and tissue clearing. The hydrophobic nature of lipids causes strong light scattering, reducing photon penetration and increasing the risk of false-negative results in fluorescence-based imaging. This limitation underscores the need for tissue clearing techniques to improve optical transparency and enhance neural visualization. However, standard organic solvent-based clearing techniques—such as benzyl alcohol/benzyl benzoate (also known as Murray’s clear [80]) and the 3DISCO (3D imaging of solvent-cleared organs) protocol [81]—can dissolve or displace adipocytes, introducing artifacts and misrepresenting fat localization. Similar issues arise during histological processing, where xylene-based dewaxing protocols for H&E staining may also alter fat distribution within tissue sections. These limitations complicate the accurate assessment of liver and pancreatic innervation in steatotic conditions, making aqueous-based clearing approaches preferable for preserving tissue integrity and achieving reliable 3D imaging.

Thus, while steatosis in the liver and pancreas primarily affects metabolic homeostasis, its impact on neurovascular architecture and histological analysis is equally significant. The interplay between fat accumulation, inflammation, and neural remodeling highlights the need for careful interpretation. This includes the use of both positive and negative labeling controls, along with multimodal imaging strategies—such as combining fluorescence imaging with stereomicroscopy and transmitted light imaging [31, 71]—to distinguish true 3D neuroanatomical patterns from artifacts.

### Debate on liver parasympathetic innervation

In the parasympathetic peripheral nervous system, both preganglionic and postganglionic neurons are considered cholinergic, meaning they primarily utilize acetylcholine as a neurotransmitter [82]. Acetylcholine is synthesized from choline and acetyl coenzyme A by the enzyme choline acetyltransferase (ChAT) and is subsequently transported into synaptic vesicles by the vesicular acetylcholine transporter (VAChT) [83]. The identification of cholinergic neurons has been essential for mapping the parasympathetic neural network, including its potential role in hepatic innervation.

To refine the specificity of cholinergic neuron detection, Arvidsson et al. [84] produced VAChT antisera for immunostaining, establishing it as a preferred marker over ChAT in neurohistology. The rationale for this choice was that ChAT is also expressed in certain non-neuronal cells, reducing its specificity for neuronal identification. Since then, VAChT has been widely recognized as a reliable marker for identifying peripheral cholinergic neurons and innervation in both animal and human studies [85]. However, the extent to which the liver receives direct parasympathetic innervation remains unresolved and subject to debate.

Two major issues underlie this uncertainty. First, although cholinergic nerve fibers have been reported in association with the human liver using histochemical staining [86, 87], this approach alone cannot establish their origin, because such fibers may derive either from parasympathetic vagal efferents or from cholinergic enteric neurons—a complexity that also applies to pancreatic innervation. Thus, VAChT labeling by itself is insufficient evidence of parasympathetic input. Anterograde tracing studies in rodents have been particularly informative in addressing this question. Using carbocyanine dyes and wheat germ agglutinin–horseradish peroxidase (WGA-HRP) tracers, Berthoud and colleagues demonstrated that vagal efferents terminate predominantly in the liver hilus, portal vein, and biliary system, and in the pancreas they project primarily to intrapancreatic ganglia [88, 89]. These findings highlight the importance of anterograde tracing for resolving the true origin of cholinergic fibers and provide a valuable reference point when evaluating the extent of parasympathetic innervation in the human liver and pancreas.

Second, the issue is further complicated by conflicting VAChT immunolabeling results. In mice, both positive [90] and negative [64, 65] reports of VAChT reactivity in the hepatic parenchyma have appeared in recent studies. In humans, 3D VAChT immunolabeling with light-sheet imaging by Adori et al. [64] and Liu et al. [65] also yielded negative findings, in contrast with earlier reports describing cholinergic nerve fibers in association with the human liver [86, 87]. These conflicting results fuel an ongoing debate about the existence and extent of direct parasympathetic innervation of the liver, which has been discussed in recent review articles [91–93]. The discrepancies may reflect not only biological variability but also technical challenges.

One major challenge is the strong autofluorescence of liver tissue (as discussed in the previous section), which interferes with VAChT signal detection and causes difficulties to distinguish true scattered cholinergic signals from background noise. Additionally, unlike the stomach and intestine—where intrinsic ganglia and neurons are well characterized in regions such as the myenteric and submucosal plexuses [94]—no intrinsic hepatic ganglia or neurons have been identified as clear landmarks for network imaging and confirmation of the VAChT labeling. This limitation is further compounded by the liver’s large volume, making comprehensive analysis more challenging. Lastly, the absence of appropriate positive controls in previous VAChT immunolabeling and imaging studies may have hindered the accurate interpretation of results. Overcoming these methodological limitations—through improved imaging, optimized immunolabeling, and rigorous use of positive controls—will be critical to clarifying the nature of liver parasympathetic innervation.

### Toward multichannel 3D high/super-resolution imaging of liver and pancreas parasympathetic innervation

To enhance the accuracy of VAChT immunolabeling and imaging, both intrinsic and extrinsic positive controls should be incorporated. Intrinsic controls involve co-staining with a second marker, such as the pan-neuronal marker PGP9.5, which highlights overall nerve tracts and fibers in the liver [71]. In this approach, VAChT-positive vesicles should spatially colocalize with PGP9.5-positive nerve extensions, confirming their neural association.

Extrinsic controls involve parallel labeling of tissues with well-established VAChT immunoreactivity, such as the stomach, gallbladder, and pancreas [19, 84, 85, 95, 96]—preferably from human samples. These reference tissues serve as benchmarks to validate the immunolabeling process and assess staining quality. If liver parasympathetic imaging yields negative results, the presence of robust VAChT signals in these control tissues would help rule out technical errors and ensure the reliability of the findings.

**Fig. 6:**
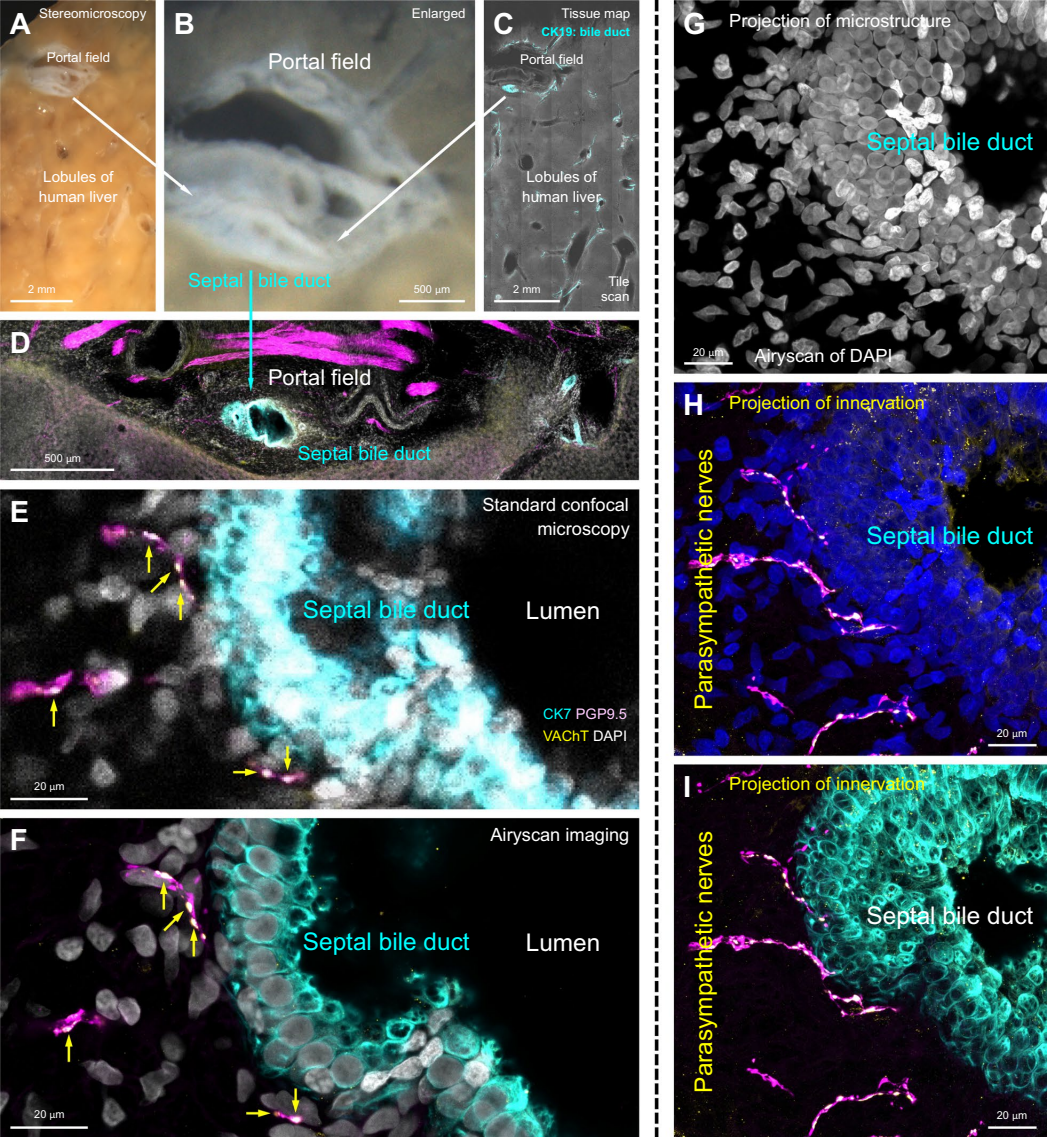
Human liver parasympathetic innervation: panoramic-to-Airyscan super-resolution imaging of septal bile duct and peri-ductal VAChT⁺ cholinergic nerves. **A–C** Stereomicroscopy and fluorescent tissue mapping of a human liver vibratome section (distal resection margin of liver hemangioma; male, 49 years old, left lobe). Cyan: CK19 labeling of bile duct epithelium; white: DAPI staining of nuclei. The portal field containing the septal bile duct is enlarged in panel **B** and further magnified in **D**. **D** Low-magnification image of the portal field. Cyan: CK19; white: DAPI; magenta: pan-neuronal marker PGP9.5; yellow: vesicular acetylcholine transporter (VAChT). The paired signals of PGP9.5 and VAChT are further magnified in panels **E** and **F**. **E, F** Standard confocal microscopy (25 × objective) followed by Airyscan imaging (40 × objective) showing VAChT⁺ parasympathetic innervation of the human liver septal bile duct. Yellow arrows indicate VAChT⁺ varicosities along PGP9.5⁺ nerve fibers. **G–I** Projection of septal bile duct microstructure and parasympathetic innervation. Signals of DAPI (nuclei: white in **G**, blue in **H**), PGP9.5 (nerves: magenta in **H**, **I**), and VAChT⁺ varicosities (yellow in **H**, **I**) were acquired using in-depth Airyscan imaging. Note that multiple rounds of fluorescence imaging were performed in this examination, which was enabled by the antifade polymer embedding (see Fig. 5) to avoid false-negative results

**Fig. 7:**
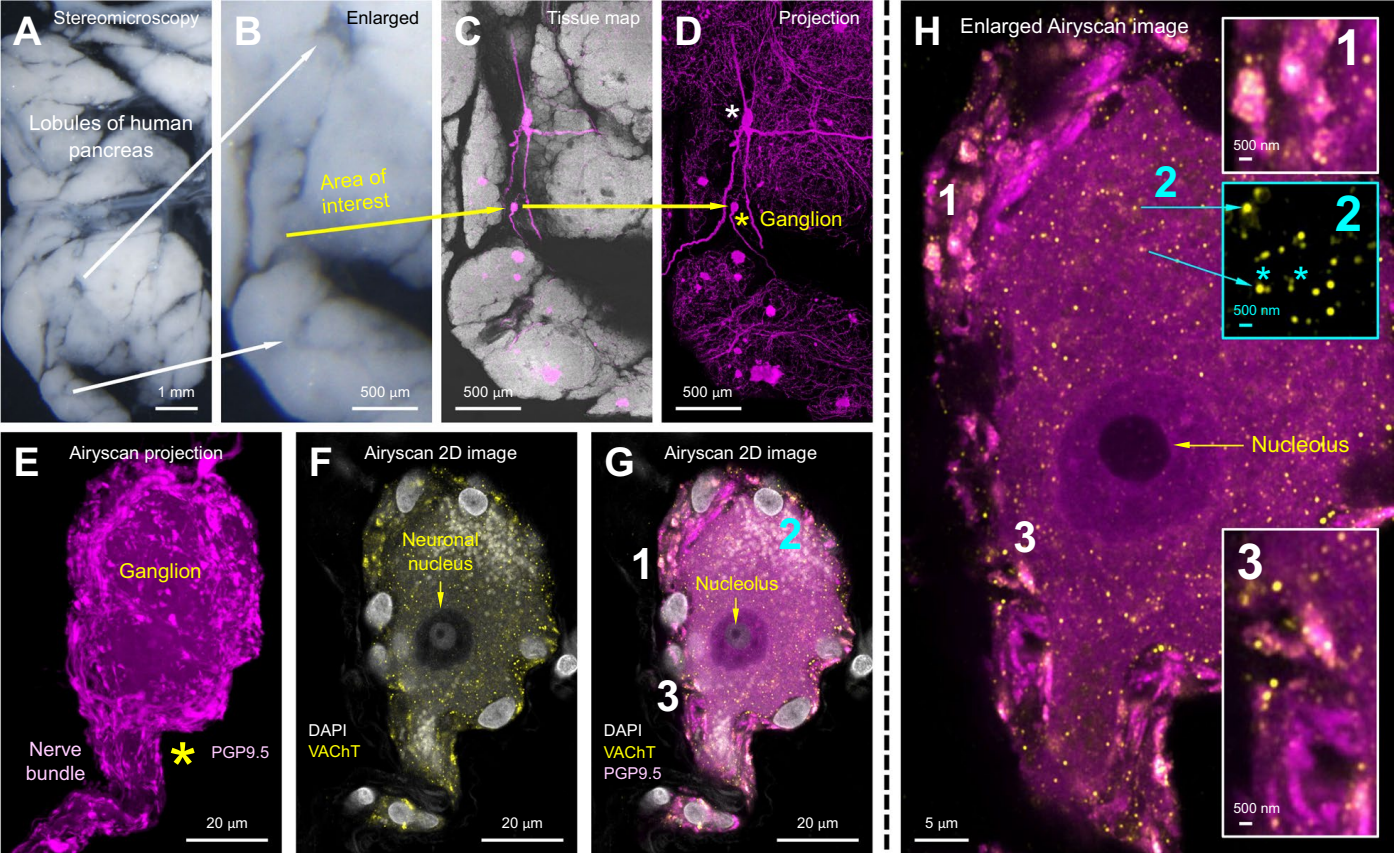
Human pancreas parasympathetic innervation: panoramic-to-Airyscan super-resolution imaging of intrapancreatic ganglion, cholinergic neuron, and VAChT⁺ vesicles. **A–D** Stereomicroscopy and fluorescent imaging of a human pancreas vibratome section (male, 49 years old, body segment of donor pancreas). Two ganglia (asterisks in panel **D**) were detected. The ganglion marked with the yellow asterisk was magnified in panels **E–G** to specify the cholinergic neuron and VAChT⁺ vesicles. **E–G** Airyscan super-resolution imaging of the cholinergic neuron. Magenta: pan-neuronal marker PGP9.5; yellow: VAChT; white: DAPI. The projection of PGP9.5 signals in **E** defines the area of the ganglion and its associated nerve bundle. DAPI signals in **F** and **G** reveal the cholinergic neuron with a dimly stained nucleus and a prominent nucleolus. Areas 1–3 in **G** are magnified in **H** to identify VAChT⁺ vesicles. **H** High-definition Airyscan image of the cholinergic neuron and VAChT⁺ vesicles. VAChT⁺ vesicles aggregate in the nerve bundles (Areas 1 and 3, insets) and soma (Area 2, inset). Asterisks in inset 2 identify two adjacent vesicles (one bright, one dim), highlighting the resolving power of the panoramic-to-Airyscan imaging approach. *Supplementary Video 2 shows the depth-resolved Airyscan super-resolution imaging of this intrapancreatic ganglion, focusing on the VAChT⁺ vesicles on Nissl bodies*

To validate VAChT staining using intrinsic and extrinsic controls, Figs 6 and 7 present paired VAChT and PGP9.5 staining to visualize human liver septal bile duct innervation, with further confirmation provided by VAChT staining of human intrapancreatic ganglia and neurons [19, 31]. Multichannel fluorescence imaging, in combination with panoramic stereomicroscopy (Figs. 6A, B**; **7A, B), tissue tile scanning (Figs. 6C, D**; **7C, D), and subsequent 3D and Airyscan super-resolution imaging (Figs. 6E–I**; **7D–H), provided sufficient resolution to visualize VAChT signals at the vesicle level. This comprehensive imaging approach revealed parasympathetic innervation specifically associated with the liver septal bile duct, using the intrinsic neurons of the pancreas as the positive control. To further enhance structural identification, CK19 was used as a secondary marker to label the hepatic ductal epithelium (Fig. 6C–F, I). Additionally, a secondary extrinsic control, the mouse gallbladder, was included to illustrate the pattern of peri-ductal parasympathetic innervation within the biliary ductal system [71].

Notably, Chen and colleagues [71] embedded liver and pancreas specimens in a UV-polymerized solid environment that excludes oxygen for antifade imaging (as discussed in Fig. 5), which effectively minimized photobleaching and reduced the risk of false-negative results during repeated rounds of high-power 3D Airyscan imaging [70, 97]. This panoramic-to-subcellular imaging approach enabled detailed characterization of the tissue microenvironment, allowing clear resolution and identification of peri-ductal VAChT-positive varicosities in the liver (Fig. 6F–I) and mapping of pancreatic innervation with vesicular structures of intrinsic neurons (Fig. 7F–H). These observations are consistent with classic anterograde tracing studies in rodents, which demonstrated vagal efferent projections to both the liver and pancreas [88, 89]. Together, these findings underscore the advantages of a multimodal, multichannel imaging strategy in detecting and confirming parasympathetic innervation in both organs with high spatial precision, while minimizing imaging artifacts.

### On focus: brain–pancreas parasympathetic network and intrapancreatic ganglia

The brain–pancreas axis is a central regulator of nutrient assimilation, coordinating islet hormone and digestive enzyme secretion in response not only to food intake but also to anticipatory sensory cues from sight, smell, and taste [98–100]. These integrated neural and hormonal signals promote efficient nutrient processing and energy storage, with glucose homeostasis as a primary outcome of this tightly regulated system.

A key effector within this axis is the parasympathetic nervous system, which initiates pancreatic islet signaling even before ingestion begins. In healthy individuals, classical physiological studies have shown that parasympathetic activity initiates the cephalic phase—a preparatory, pre-absorptive response triggered by external food-related stimuli that activates neuronal and pancreatic hormone signaling [101–103]. In a landmark study, Ahren and Holst [104] demonstrated that this early-phase insulin release significantly contributes to postprandial glucose regulation, involving both vagally mediated cholinergic and noncholinergic mechanisms.

Once sensory information is processed in the brainstem, vagal efferent signals descend through parasympathetic pathways and relay to intrapancreatic ganglia—key neural hubs embedded within pancreatic lobules that innervate both the endocrine and exocrine compartments [3, 4, 19, 31, 105]. These ganglia coordinate parasympathetic outflow to the estimated 1–2 million islets scattered throughout the human pancreas, initiating insulin secretion (the cephalic phase). As digestion progresses, β-cell stimulation is further enhanced by circulating incretin hormones such as GLP-1 [11, 106, 107], along with rising blood glucose levels (the dominant physiological trigger). These combined signals generate a robust postprandial insulin response, facilitating systemic glucose uptake and restoring euglycemia.

While GLP-1–based therapies, which act through the endocrine/circulatory limb of this regulatory axis, are now widely used to treat type 2 diabetes and obesity [108], the neural pathway remains underdeveloped in clinical care. This underrepresentation reflects not a lack of physiological importance, but the technical challenges of mapping and analyzing the pancreas’s complex neural architecture. Among the most critical—and least characterized—components are the intrapancreatic ganglia, which serve as integration hubs for parasympathetic signaling and modulators of islet hormone output.

Unlocking the therapeutic potential of this neuroendocrine circuit requires detailed mapping of the cellular composition, neurochemical phenotype, and spatial connectivity of intrapancreatic ganglia. As described in earlier sections, 3D and Airyscan super-resolution imaging overcomes the limitations of conventional 2D histology by providing vesicle-level resolution in intact pancreatic tissues [70–72]. This depth-resolved approach enables high-fidelity visualization of intrapancreatic ganglia—including neurons, glial-neuronal interactions, and associated vasculature—within their native microenvironment (**Supplementary Videos 2** and **3**). To support this level of structural insight, high-refractive-index polymer embedding (Fig. 5) provides optical clarity and preserves fluorophores during extended imaging sessions. This anatomically precise and scalable platform enables consistent mapping and characterization of intrapancreatic ganglia, supporting the development of neuromodulatory strategies for metabolic diseases involving islet dysfunction.

## Future directions and emerging opportunities

Recent advancements in 3D neurohistological imaging and tissue processing have provided new insights into the complex innervation patterns of the human liver and pancreas. Moving forward, integrating these innovative techniques with emerging imaging modalities—such as adaptive optics [109], expansion microscopy [110], and correlative light and electron microscopy [111]—will be essential for achieving greater spatial resolution and sensitivity in neuroanatomical studies. However, several key challenges remain, and further progress in this field will require interdisciplinary approaches that refine imaging methods, improve data validation, and enhance clinical applicability.

To build upon recent advancements, future research on human liver and pancreas innervation and its role in metabolic regulation could benefit from the following areas of focus:

- *Refinement of imaging techniques* Continued development of multimodal imaging strategies that integrate panoramic stereomicroscopy, tile scanning, and super-resolution methods (e.g., detector array microscopy, structured illumination microscopy, and stimulated emission depletion microscopy) will enable comprehensive mapping of neural networks at both macro and micro scales. Further emphasis on minimizing artifacts—such as autofluorescence and photobleaching—through improved chemical treatments and optimized imaging protocols will enhance image fidelity and reproducibility.
- *Signal validation* Ensuring the accuracy of neuroanatomical findings will require the systematic use of intrinsic and extrinsic positive controls in immunolabeling. Cross-validation with complementary imaging techniques will be particularly critical for confirming the presence of liver parasympathetic innervation, an area where conflicting reports persist.
- *Clinical translation and pathophysiological insights* Since animal models, particularly rodents, do not fully recapitulate the structural and functional complexities of the human liver and pancreas, direct correlation between neuroanatomical findings and clinical outcomes is essential. A deeper understanding of the interplay between neural control and metabolic dysregulation could lead to the development of novel diagnostic markers and therapeutic strategies for metabolic disorders.
- *Integration of multichannel and multiscale data* The convergence of high-resolution imaging data with computational tools, such as machine learning and 3D reconstruction algorithms, has the potential to generate detailed, quantitative features of hepatic and pancreatic innervation. These integrative approaches will aid in detecting subtle neurovascular remodeling and enable high-resolution mapping of cellular innervation patterns—such as islet cell innervation—across both healthy and diseased states.

Ultimately, the future of human liver and pancreas neurohistology depends on leveraging these technological advancements not only to overcome current methodological challenges but also to reveal previously unrecognized neuroanatomical features and regulatory mechanisms. These efforts will pave the way for a more comprehensive understanding of metabolic homeostasis and its dysregulation in disease states, with implications for both fundamental research and clinical applications.

## Conclusions

This review has highlighted the critical role of the liver and pancreas in metabolic regulation, emphasizing the importance of accurately mapping their intricate neural networks. We have discussed the significant challenges posed by factors such as tissue autofluorescence, autolysis, photobleaching, and steatosis, all of which complicate 3D immunohistochemical imaging. Advances in autofluorescence reduction, tissue clearing, and the development of robust sample preparation and imaging protocols—coupled with the integration of both intrinsic and extrinsic controls—have markedly improved our ability to visualize and interpret neural structures in the human liver and pancreas specimens.

Key findings include the successful application of multichannel imaging strategies to resolve parasympathetic innervation in the liver and pancreas, as well as the use of antifade polymer embedding to mitigate photobleaching and preserve fine neural details in 3D. These methodological advances provide a strong foundation for ongoing investigations into liver parasympathetic innervation and the intricate parasympathetic network of the pancreas—particularly the structural connectivity and neurochemical identities of neurons within intrapancreatic ganglia. The structural connectivity supports the role of these ganglia as central hubs linking parasympathetic input to downstream targets, while the neurochemical identities offer molecular specificity for understanding neural control of islet function and guiding the development of neuromodulatory therapies for metabolic disease.

In summary, overcoming the technical challenges inherent in 3D neurohistology has opened new avenues for exploring the neural regulation of metabolic functions. As imaging techniques continue to advance, they will not only refine our understanding of normal physiology but also reveal the neural alterations underlying metabolic diseases. The integration of innovative imaging approaches with rigorous validation methods promises to drive the field forward, ultimately leading to improved diagnostic and therapeutic strategies in clinical settings.

## Supplementary Information

Additional file 1.

Additional file 2.

Additional file 3.

Additional file 4.

Additional file 5.

## Data Availability

The image data of this review are available from the corresponding author on reasonable request.

## Abbreviations

2D: 2-Dimensional
3D: 3-Dimensional
A-ha polymer: Poly(acrylamide-*co*–N-hydroxymethyl acrylamide)
AF: Alexa fluor
ChAT: Choline acetyltransferase
CK19: Cytokeratin 19
GLP-1: Glucagon-like peptide-1
H&E: Hematoxylin and eosin
LED: Light-emitting diode
*n*: Refractive index
NAFLD: Non-alcoholic fatty liver disease
PanIN: Pancreatic intraepithelial neoplasia
ROS: Reactive oxygen species
STED microscopy: Stimulated emission depletion microscopy
UV: Ultraviolet radiation
VAChT: Vesicular acetylcholine transporter

## References

[1] Yi CX, la Fleur SE, Fliers E, Kalsbeek A. The role of the autonomic nervous liver innervation in the control of energy metabolism. Biochim Biophys Acta. 2010;1802(4):416–31. 20060897 10.1016/j.bbadis.2010.01.006

[2] Kandilis AN, Papadopoulou IP, Koskinas J, Sotiropoulos G, Tiniakos DG. Liver innervation and hepatic function: new insights. J Surg Res. 2015;194(2):511–9. 25555404 10.1016/j.jss.2014.12.006

[3] Berthoud HR. Anatomy and function of sensory hepatic nerves. Anat Rec A Discov Mol Cell Evol Biol. 2004;280(1):827–35. 15382018 10.1002/ar.a.20088

[4] Ahren B. Autonomic regulation of islet hormone secretion–implications for health and disease. Diabetologia. 2000;43(4):393–410. 10819232 10.1007/s001250051322

[5] Hampton RF, Jimenez-Gonzalez M, Stanley SA. Unravelling innervation of pancreatic islets. Diabetologia. 2022;65(7):1069–84. 35348820 10.1007/s00125-022-05691-9 PMC9205575

[6] Taborsky GJ Jr, Mundinger TO. Minireview: The role of the autonomic nervous system in mediating the glucagon response to hypoglycemia. Endocrinology. 2012;153(3):1055–62. 22315452 10.1210/en.2011-2040 PMC3384078

[7] Vinik AI, Maser RE, Mitchell BD, Freeman R. Diabetic autonomic neuropathy. Diabetes Care. 2003;26(5):1553–79. 12716821 10.2337/diacare.26.5.1553

[8] McCall AL. Insulin therapy and hypoglycemia. Endocrinol Metab Clin North Am. 2012;41(1):57–87. 22575407 10.1016/j.ecl.2012.03.001 PMC4265808

[9] Wehrwein EA, Orer HS, Barman SM. Overview of the anatomy, physiology, and pharmacology of the autonomic nervous system. Compr Physiol. 2016;6(3):1239–78. 27347892 10.1002/cphy.c150037

[10] Goldstein DS. Adrenal responses to stress. Cell Mol Neurobiol. 2010;30(8):1433–40. 21061156 10.1007/s10571-010-9606-9 PMC3056281

[11] Drucker DJ. The biology of incretin hormones. Cell Metab. 2006;3(3):153–65. 16517403 10.1016/j.cmet.2006.01.004

[12] Gilon P, Henquin JC. Mechanisms and physiological significance of the cholinergic control of pancreatic beta-cell function. Endocr Rev. 2001;22(5):565–604. 11588141 10.1210/edrv.22.5.0440

[13] Miller BM, Oderberg IM, Goessling W. Hepatic nervous system in development, regeneration, and disease. Hepatology. 2021;74(6):3513–22. 34256416 10.1002/hep.32055 PMC8639644

[14] Uyama N, Geerts A, Reynaert H. Neural connections between the hypothalamus and the liver. Anat Rec A Discov Mol Cell Evol Biol. 2004;280(1):808–20. 15382020 10.1002/ar.a.20086

[15] Fukuda Y, Imoto M, Koyama Y, Miyazawa Y, Hayakawa T. Demonstration of noradrenaline-immunoreactive nerve fibres in the liver. J Int Med Res. 1996;24(6):466–72. 8959530 10.1177/030006059602400603

[16] Tsuneki K, Ichihara K. Electron microscope study of vertebrate liver innervation. Arch Histol Jpn. 1981;44(1):1–13. 7316686 10.1679/aohc1950.44.1

[17] Smits MM, van Geenen EJ. The clinical significance of pancreatic steatosis. Nat Rev Gastroenterol Hepatol. 2011;8(3):169–77. 21304475 10.1038/nrgastro.2011.4

[18] Ramkissoon R, Gardner TB. Pancreatic steatosis: an emerging clinical entity. Am J Gastroenterol. 2019;114(11):1726–34. 31185002 10.14309/ajg.0000000000000262

[19] Tang SC, Baeyens L, Shen CN, Peng SJ, Chien HJ, Scheel DW, et al. Human pancreatic neuro-insular network in health and fatty infiltration. Diabetologia. 2018;61(1):168–81. 28852792 10.1007/s00125-017-4409-x

[20] Rebours V, Gaujoux S, d’Assignies G, Sauvanet A, Ruszniewski P, Levy P, et al. Obesity and fatty pancreatic infiltration are risk factors for pancreatic precancerous lesions (PanIN). Clin Cancer Res. 2015;21(15):3522–8. 25700304 10.1158/1078-0432.CCR-14-2385

[21] Schaefer PM, Kalinina S, Rueck A, von Arnim CAF, von Einem B. NADH autofluorescence-a marker on its way to boost bioenergetic research. Cytometry A. 2019;95(1):34–46. 30211978 10.1002/cyto.a.23597

[22] Wallrabe H, Svindrych Z, Alam SR, Siller KH, Wang T, Kashatus D, et al. Segmented cell analyses to measure redox states of autofluorescent NAD(P)H, FAD & Trp in cancer cells by FLIM. Sci Rep. 2018;8(1):79. 29311591 10.1038/s41598-017-18634-x PMC5758727

[23] Croce AC, Ferrigno A, Bottiroli G, Vairetti M. Autofluorescence-based optical biopsy: an effective diagnostic tool in hepatology. Liver Int. 2018;38(7):1160–74. 29624848 10.1111/liv.13753

[24] Croce AC, Ferrigno A, Santin G, Vairetti M, Bottiroli G. Bilirubin: an autofluorescence bile biomarker for liver functionality monitoring. J Biophotonics. 2014;7(10):810–7. 23616471 10.1002/jbio.201300039

[25] Schnell SA, Staines WA, Wessendorf MW. Reduction of lipofuscin-like autofluorescence in fluorescently labeled tissue. J Histochem Cytochem. 1999;47(6):719–30. 10330448 10.1177/002215549904700601

[26] Di Guardo G. Lipofuscin, lipofuscin-like pigments and autofluorescence. Eur J Histochem. 2015;59(1):2485. 25820564 10.4081/ejh.2015.2485 PMC4378218

[27] Ueda Y, Kobayashi M. Spectroscopic studies of autofluorescence substances existing in human tissue: influences of lactic acid and porphyrins. Appl Opt. 2004;43(20):3993–8. 15285088 10.1364/ao.43.003993

[28] Shrirao AB, Schloss RS, Fritz Z, Shrirao MV, Rosen R, Yarmush ML. Autofluorescence of blood and its application in biomedical and clinical research. Biotechnol Bioeng. 2021;118(12):4550–76. 34487351 10.1002/bit.27933

[29] Baschong W, Suetterlin R, Laeng RH. Control of autofluorescence of archival formaldehyde-fixed, paraffin-embedded tissue in confocal laser scanning microscopy (CLSM). J Histochem Cytochem. 2001;49(12):1565–72. 11724904 10.1177/002215540104901210

[30] Erben T, Ossig R, Naim HY, Schnekenburger J. What to do with high autofluorescence background in pancreatic tissues - an efficient Sudan black B quenching method for specific immunofluorescence labelling. Histopathology. 2016;69(3):406–22. 26802460 10.1111/his.12935

[31] Chien HJ, Chiang TC, Peng SJ, Chung MH, Chou YH, Lee CY, et al. Human pancreatic afferent and efferent nerves: mapping and 3-D illustration of exocrine, endocrine, and adipose innervation. Am J Physiol Gastrointest Liver Physiol. 2019;317(5):G694–706. 31509431 10.1152/ajpgi.00116.2019

[32] Chung MH, Chien HJ, Peng SJ, Chou YH, Chiang TC, Chang HP, et al. Multimodal 3-D/2-D human islet and duct imaging in exocrine and endocrine lesion environment: associated pancreas tissue remodeling. Am J Physiol Endocrinol Metab. 2022;323(4):E354–65. 35947703 10.1152/ajpendo.00111.2022

[33] Sakr N, Glazova O, Shevkova L, Onyanov N, Kaziakhmedova S, Shilova A, et al. Characterizing and quenching autofluorescence in fixed mouse adrenal cortex tissue. Int J Mol Sci. 2023;24(4):3432. 36834842 10.3390/ijms24043432 PMC9968082

[34] Zhang Z, Fan H, Richardson W, Gao BZ, Ye T. Management of autofluorescence in formaldehyde-fixed myocardium: choosing the right treatment. Eur J Histochem. 2023;67(4):3812. 37781779 10.4081/ejh.2023.3812 PMC10614721

[35] Oliveira VC, Carrara RC, Simoes DL, Saggioro FP, Carlotti CG Jr, Covas DT, et al. Sudan Black B treatment reduces autofluorescence and improves resolution of in situ hybridization specific fluorescent signals of brain sections. Histol Histopathol. 2010;25(8):1017–24. 20552552 10.14670/HH-25.1017

[36] Sun Y, Yu H, Zheng D, Cao Q, Wang Y, Harris D. Sudan black B reduces autofluorescence in murine renal tissue. Arch Pathol Lab Med. 2011;135(10):1335–42. 21970489 10.5858/arpa.2010-0549-OA

[37] Radtke AJ, Chu CJ, Yaniv Z, Yao L, Marr J, Beuschel RT, et al. IBEX: an iterative immunolabeling and chemical bleaching method for high-content imaging of diverse tissues. Nat Protoc. 2022;17(2):378–401. 35022622 10.1038/s41596-021-00644-9 PMC13430508

[38] Liu CH, Lin CH, Tsai MJ, Chen WT, Chai CY, Huang YC, et al. Melanin bleaching with dilute hydrogen peroxide: a simple and rapid method. Appl Immunohistochem Mol Morphol. 2013;21(3):275–9. 23060296 10.1097/PAI.0b013e31826d81db

[39] Chung JY, Choi J, Sears JD, Ylaya K, Perry C, Choi CH, et al. A melanin-bleaching methodology for molecular and histopathological analysis of formalin-fixed paraffin-embedded tissue. Lab Invest. 2016;96(10):1116–27. 27548802 10.1038/labinvest.2016.90 PMC7781076

[40] Duong H, Han M. A multispectral LED array for the reduction of background autofluorescence in brain tissue. J Neurosci Methods. 2013;220(1):46–54. 23994358 10.1016/j.jneumeth.2013.08.018 PMC3856220

[41] Nolta NF, Liberti A, Makol R, Han M. Gelatin embedding and LED autofluorescence reduction for rodent spinal cord histology. J Neurosci Methods. 2020;1(346):108924. 10.1016/j.jneumeth.2020.108924 PMC7606419 32918967

[42] Zheng J, Wu YC, Phillips EH, Cai X, Wang X, Seung-Young Lee S. Increased multiplexity in optical tissue clearing-based three-dimensional immunofluorescence microscopy of the tumor microenvironment by light-emitting diode photobleaching. Lab Invest. 2024;104(6):102072. 38679160 10.1016/j.labinv.2024.102072 PMC11240282

[43] Ueda HR, Erturk A, Chung K, Gradinaru V, Chedotal A, Tomancak P, et al. Tissue clearing and its applications in neuroscience. Nat Rev Neurosci. 2020;21(2):61–79. 31896771 10.1038/s41583-019-0250-1 PMC8121164

[44] Richardson DS, Guan W, Matsumoto K, Pan C, Chung K, Erturk A, et al. Tissue clearing. Nat Rev Methods Primers. 2021. 10.1038/s43586-021-00080-9. 35128463 10.1038/s43586-021-00080-9 PMC8815095

[45] Tainaka K, Kuno A, Kubota SI, Murakami T, Ueda HR. Chemical principles in tissue clearing and staining protocols for whole-body cell profiling. Annu Rev Cell Dev Biol. 2016;6(32):713–41. 10.1146/annurev-cellbio-111315-125001 27298088

[46] Holtzer RL, Van Lancker JL. Early changes in pancreas autolysis. Am J Pathol. 1962;40(3):331–6. 13908608 PMC1949508

[47] Shimizu M, Hayashi T, Saitoh Y, Ohta K, Itoh H. Postmortem autolysis in the pancreas: multivariate statistical study. The influence of clinicopathological conditions. Pancreas. 1990;5(1):91–4. 2293713 10.1097/00006676-199001000-00013

[48] Walker AE. The adult pancreas in trauma and disease. Acad Forensic Pathol. 2018;8(2):192–218. 31240039 10.1177/1925362118781612 PMC6490126

[49] Alwelaie Y, Point du Jour KS, Pandya S, Goodman AL, Centeno BA, Adsay V, et al. Acinar cell induced autolysis is a frequent occurrence in CytoLyt-fixed pancreatic fine needle aspiration specimens: an analysis of 157 cytology samples. Cancer Cytopathol. 2021;129(4):283–90. 33136337 10.1002/cncy.22378

[50] Qadir MMF, Alvarez-Cubela S, Weitz J, Panzer JK, Klein D, Moreno-Hernandez Y, et al. Long-term culture of human pancreatic slices as a model to study real-time islet regeneration. Nat Commun. 2020;11(1):3265. 32601271 10.1038/s41467-020-17040-8 PMC7324563

[51] Alver CG, Alvarez-Cubela S, Altilio I, Hutchison E, Warrner E, Viso ME, et al. SliceChip: a benchtop fluidic platform for organotypic culture and serial assessment of human and rodent pancreatic slices. Lab Chip. 2024;24(6):1557–72. 38205530 10.1039/d3lc00850a PMC10939771

[52] Monici M. Cell and tissue autofluorescence research and diagnostic applications. Biotechnol Annu Rev. 2005;11:227–56. 16216779 10.1016/S1387-2656(05)11007-2

[53] DiMaio VJ, DiMaio D. Forensic pathology. 2nd ed. Boca Raton: CRC Press; 2001.

[54] Demchenko AP. Photobleaching of organic fluorophores: quantitative characterization, mechanisms, protection. Methods Appl Fluoresc. 2020;8(2):022001. 32028269 10.1088/2050-6120/ab7365

[55] Bernas T, Zarebski M, Dobrucki JW, Cook PR. Minimizing photobleaching during confocal microscopy of fluorescent probes bound to chromatin: role of anoxia and photon flux. J Microsc. 2004;215(Pt 3):281–96. 15312193 10.1111/j.0022-2720.2004.01377.x

[56] Kwon J, Elgawish MS, Shim SH. Bleaching-resistant super-resolution fluorescence microscopy. Adv Sci (Weinh). 2022;9(9):e2101817. 35088584 10.1002/advs.202101817 PMC8948665

[57] Patterson GH, Piston DW. Photobleaching in two-photon excitation microscopy. Biophys J. 2000;78(4):2159–62. 10733993 10.1016/S0006-3495(00)76762-2 PMC1300807

[58] Dasgupta A, Koerfer A, Kokot B, Urbancic I, Eggeling C, Carravilla P. Effects and avoidance of photoconversion-induced artifacts in confocal and STED microscopy. Nat Methods. 2024;21(7):1171–4. 38834747 10.1038/s41592-024-02297-4 PMC11543600

[59] Haugland RP. The handbook: a guide to fluorescent probes and labeling technologies. 10th ed. Waltham: Invitrogen Corp; 2005.

[60] Aitken CE, Marshall RA, Puglisi JD. An oxygen scavenging system for improvement of dye stability in single-molecule fluorescence experiments. Biophys J. 2008;94(5):1826–35. 17921203 10.1529/biophysj.107.117689 PMC2242739

[61] Huisken J, Swoger J, Del Bene F, Wittbrodt J, Stelzer EH. Optical sectioning deep inside live embryos by selective plane illumination microscopy. Science. 2004;305(5686):1007–9. 15310904 10.1126/science.1100035

[62] Keller PJ, Schmidt AD, Wittbrodt J, Stelzer EH. Reconstruction of zebrafish early embryonic development by scanned light sheet microscopy. Science. 2008;322(5904):1065–9. 18845710 10.1126/science.1162493

[63] Power RM, Huisken J. A guide to light-sheet fluorescence microscopy for multiscale imaging. Nat Methods. 2017;14(4):360–73. 28362435 10.1038/nmeth.4224

[64] Adori C, Daraio T, Kuiper R, Barde S, Horvathova L, Yoshitake T, et al. Disorganization and degeneration of liver sympathetic innervations in nonalcoholic fatty liver disease revealed by 3D imaging. Sci Adv. 2021. 10.1126/sciadv.abg5733. 34290096 10.1126/sciadv.abg5733 PMC8294768

[65] Liu K, Yang L, Wang G, Liu J, Zhao X, Wang Y, et al. Metabolic stress drives sympathetic neuropathy within the liver. Cell Metab. 2021;33(3):666-75 e4. 33545051 10.1016/j.cmet.2021.01.012

[66] Durgam S, Singh B, Cole SL, Brokken MT, Stewart M. Quantitative assessment of tendon hierarchical structure by combined second harmonic generation and immunofluorescence microscopy. Tissue Eng Part C Methods. 2020;26(5):253–62. 32228165 10.1089/ten.TEC.2020.0032

[67] Chapman KB, Filipsky F, Peschke N, Gelleri M, Weinhardt V, Braun A, et al. A comprehensive method to study the DNA’s association with Lamin And Chromatin compaction in intact cell nuclei at super resolution. Nanoscale. 2023;15(2):742–56. 36524744 10.1039/d2nr02684h PMC9813922

[68] Florijn RJ, Slats J, Tanke HJ, Raap AK. Analysis of antifading reagents for fluorescence microscopy. Cytometry. 1995;19(2):177–82. 7743897 10.1002/cyto.990190213

[69] Arsic A, Stajkovic N, Spiegel R, Nikic-Spiegel I. Effect of Vectashield-induced fluorescence quenching on conventional and super-resolution microscopy. Sci Rep. 2020;10(1):6441. 32296095 10.1038/s41598-020-63418-5 PMC7160131

[70] Hsiao FT, Chien HJ, Chou YH, Peng SJ, Chung MH, Huang TH, et al. Transparent tissue in solid state for solvent-free and antifade 3D imaging. Nat Commun. 2023;14(1):3395. 37296117 10.1038/s41467-023-39082-4 PMC10256715

[71] Chen CC, Peng SJ, Chou YH, Lee CY, Lee PH, Hu RH, et al. Human liver afferent and efferent nerves revealed by 3-D/Airyscan super-resolution imaging. Am J Physiol Endocrinol Metab. 2024;326(2):E107–23. 38170164 10.1152/ajpendo.00205.2023

[72] Lee CY, Kuo TC, Chou YH, Peng SJ, Hsiao FT, Chung MH, et al. 3D imaging resolves human pancreatic duct-beta-cell clusters during cystic change. Diabetes. 2025;74(5):734–48. 39787388 10.2337/db24-0824 PMC12015146

[73] ZEISS Lattice SIM 5. Resolve the details hiding in the depth. https://www.zeiss.com/microscopy/us/products/light-microscopes/super-resolution-microscopes/lattice-sim-5.html. Accessed 30 September 2025.

[74] Nikon Instruments Inc. Application note: precision imaging in complex tissue Structures. https://www.microscope.healthcare.nikon.com/resources/application-notes/precision-imaging-in-complex-tissue-structures. Accessed 30 September 2025.

[75] Farrell GC, Larter CZ. Nonalcoholic fatty liver disease: from steatosis to cirrhosis. Hepatology. 2006;43(2 Suppl 1):S99–112. 16447287 10.1002/hep.20973

[76] Burt AD, Mutton A, Day CP. Diagnosis and interpretation of steatosis and steatohepatitis. Semin Diagn Pathol. 1998;15(4):246–58. 9845426

[77] Tilg H, Moschen AR. Evolution of inflammation in nonalcoholic fatty liver disease: the multiple parallel hits hypothesis. Hepatology. 2010;52(5):1836–46. 21038418 10.1002/hep.24001

[78] Pavlov VA, Tracey KJ. The vagus nerve and the inflammatory reflex–linking immunity and metabolism. Nat Rev Endocrinol. 2012;8(12):743–54. 23169440 10.1038/nrendo.2012.189 PMC4082307

[79] Tien YW, Chien HJ, Chiang TC, Chung MH, Lee CY, Peng SJ, et al. Local islet remodelling associated with duct lesion-islet complex in adult human pancreas. Diabetologia. 2021;64(10):2266–78. 34272581 10.1007/s00125-021-05504-5

[80] Dodt HU, Leischner U, Schierloh A, Jahrling N, Mauch CP, Deininger K, et al. Ultramicroscopy: three-dimensional visualization of neuronal networks in the whole mouse brain. Nat Methods. 2007;4(4):331–6. 17384643 10.1038/nmeth1036

[81] Erturk A, Becker K, Jahrling N, Mauch CP, Hojer CD, Egen JG, et al. Three-dimensional imaging of solvent-cleared organs using 3DISCO. Nat Protoc. 2012;7(11):1983–95. 23060243 10.1038/nprot.2012.119

[82] Kandel ER, Schwartz JH, Jessell TM, Siegelbaum SA, Hudspeth AJ. Principles of neural science. 5th ed. Columbus: McGraw-Hill; 2013.

[83] Usdin TB, Eiden LE, Bonner TI, Erickson JD. Molecular biology of the vesicular ACh transporter. Trends Neurosci. 1995;18(5):218–24. 7610492 10.1016/0166-2236(95)93906-e

[84] Arvidsson U, Riedl M, Elde R, Meister B. Vesicular acetylcholine transporter (VAChT) protein: a novel and unique marker for cholinergic neurons in the central and peripheral nervous systems. J Comp Neurol. 1997;378(4):454–67. 9034903

[85] Schafer MK, Eiden LE, Weihe E. Cholinergic neurons and terminal fields revealed by immunohistochemistry for the vesicular acetylcholine transporter. II. The peripheral nervous system. Neuroscience. 1998;84(2):361–76. 9539210 10.1016/s0306-4522(97)80196-0

[86] Amenta F, Cavallotti C, Ferrante F, Tonelli F. Cholinergic nerves in the human liver. Histochem J. 1981;13(3):419–24. 7251394 10.1007/BF01005057

[87] Akiyoshi H, Gonda T, Terada T. A comparative histochemical and immunohistochemical study of aminergic, cholinergic and peptidergic innervation in rat, hamster, guinea pig, dog and human livers. Liver. 1998;18(5):352–9. 9831365 10.1111/j.1600-0676.1998.tb00817.x

[88] Berthoud HR, Kressel M, Neuhuber WL. An anterograde tracing study of the vagal innervation of rat liver, portal vein and biliary system. Anat Embryol (Berl). 1992;186(5):431–42. 1280009 10.1007/BF00185458

[89] Berthoud HR, Powley TL. Morphology and distribution of efferent vagal innervation of rat pancreas as revealed with anterograde transport of Dil. Brain Res. 1991;553(2):336–41. 1718546 10.1016/0006-8993(91)90846-n

[90] Hwang J, Okada J, Liu L, Pessin JE, Schwartz GJ, Jo YH. The development of hepatic steatosis depends on the presence of liver-innervating parasympathetic cholinergic neurons in mice fed a high-fat diet. PLoS Biol. 2024;22(10):e3002865. 39436946 10.1371/journal.pbio.3002865 PMC11530026

[91] Metz CN, Pavlov VA. Vagus nerve cholinergic circuitry to the liver and the gastrointestinal tract in the neuroimmune communicatome. Am J Physiol Gastrointest Liver Physiol. 2018;315(5):G651–8. 30001146 10.1152/ajpgi.00195.2018 PMC6293249

[92] Berthoud HR, Munzberg H, Morrison CD, Neuhuber WL. Hepatic interoception in health and disease. Auton Neurosci. 2024;253:103174. 38579493 10.1016/j.autneu.2024.103174 PMC11129274

[93] Berthoud HR, Fox EA, Munzberg H, Yu S, Kim A, Lowell BB, et al. Direct vagal input to the gastrointestinal tract and other viscera: Re-definition of autonomic neuroscience or experimental artifacts? Auton Neurosci. 2025;260:103310. 40499395 10.1016/j.autneu.2025.103310 PMC12362343

[94] Furness JB. The enteric nervous system and neurogastroenterology. Nat Rev Gastroenterol Hepatol. 2012;9(5):286–94. 22392290 10.1038/nrgastro.2012.32

[95] Li ZS, Fox-Threlkeld JE, Furness JB. Innervation of intestinal arteries by axons with immunoreactivity for the vesicular acetylcholine transporter (VAChT). J Anat. 1998;192(Pt 1):107–17. 9568566 10.1046/j.1469-7580.1998.19210107.x PMC1467744

[96] Tang SC, Shen CN, Lin PY, Peng SJ, Chien HJ, Chou YH, et al. Pancreatic neuro-insular network in young mice revealed by 3d panoramic histology. Diabetologia. 2018;61(1):158–67. 28864913 10.1007/s00125-017-4408-y

[97] Huff J. The Airyscan detector from ZEISS: confocal imaging with improved signal-to-noise ratio and super-resolution. Nature Methods. 2015 Dec;12(12).

[98] Begg DP, Woods SC. Interactions between the central nervous system and pancreatic islet secretions: a historical perspective. Adv Physiol Educ. 2013;37(1):53–60. 23471249 10.1152/advan.00167.2012 PMC3776474

[99] Lkhagvasuren B, Mee-Inta O, Zhao ZW, Hiramoto T, Boldbaatar D, Kuo YM. Pancreas-brain crosstalk. Front Neuroanat. 2021;15:691777. 34354571 10.3389/fnana.2021.691777 PMC8329585

[100] Rosario W, Singh I, Wautlet A, Patterson C, Flak J, Becker TC, et al. The brain-to-pancreatic islet neuronal map reveals differential glucose regulation from distinct hypothalamic regions. Diabetes. 2016;65(9):2711–23. 27207534 10.2337/db15-0629 PMC5001176

[101] Teff K. Nutritional implications of the cephalic-phase reflexes: endocrine responses. Appetite. 2000;34(2):206–13. 10744911 10.1006/appe.1999.0282

[102] Teff KL. Cephalic phase pancreatic polypeptide responses to liquid and solid stimuli in humans. Physiol Behav. 2010;99(3):317–23. 19944113 10.1016/j.physbeh.2009.11.009 PMC2834473

[103] Smeets PA, Erkner A, de Graaf C. Cephalic phase responses and appetite. Nutr Rev. 2010;68(11):643–55. 20961295 10.1111/j.1753-4887.2010.00334.x

[104] Ahren B, Holst JJ. The cephalic insulin response to meal ingestion in humans is dependent on both cholinergic and noncholinergic mechanisms and is important for postprandial glycemia. Diabetes. 2001;50(5):1030–8. 11334405 10.2337/diabetes.50.5.1030

[105] Ahren B. Islet nerves in focus–defining their neurobiological and clinical role. Diabetologia. 2012;55(12):3152–4. 23001378 10.1007/s00125-012-2727-6

[106] Nauck MA, Meier JJ. Incretin hormones: their role in health and disease. Diabetes Obes Metab. 2018;20(Suppl 1):5–21. 29364588 10.1111/dom.13129

[107] Drucker DJ, Habener JF, Holst JJ. Discovery, characterization, and clinical development of the glucagon-like peptides. J Clin Invest. 2017;127(12):4217–27. 29202475 10.1172/JCI97233 PMC5707151

[108] Drucker DJ. GLP-1-based therapies for diabetes, obesity and beyond. Nat Rev Drug Discov. 2025. 10.1038/s41573-025-01183-8 40281304

[109] Booth MJ. Adaptive optics in microscopy. Philos Trans A Math Phys Eng Sci. 1861;2007(365):2829–43. 10.1098/rsta.2007.0013 17855218

[110] Wassie AT, Zhao Y, Boyden ES. Expansion microscopy: principles and uses in biological research. Nat Methods. 2019;16(1):33–41. 30573813 10.1038/s41592-018-0219-4 PMC6373868

[111] Bykov YS, Cortese M, Briggs JA, Bartenschlager R. Correlative light and electron microscopy methods for the study of virus-cell interactions. FEBS Lett. 2016;590(13):1877–95. 27008928 10.1002/1873-3468.12153

