# Supplementary material for: Human liver and pancreas innervation: resolving 3D neurohistological challenges and advancing insights
Source: J Biomed Sci. 2025 Nov 10;32:97. doi: 10.1186/s12929-025-01194-y (PMC12599030; doi:10.1186/s12929-025-01194-y)
Supplement: Supplementary file 1 — Additional file 1. [file 12929_2025_1194_MOESM1_ESM.pdf]

Supplementary Fig. 1

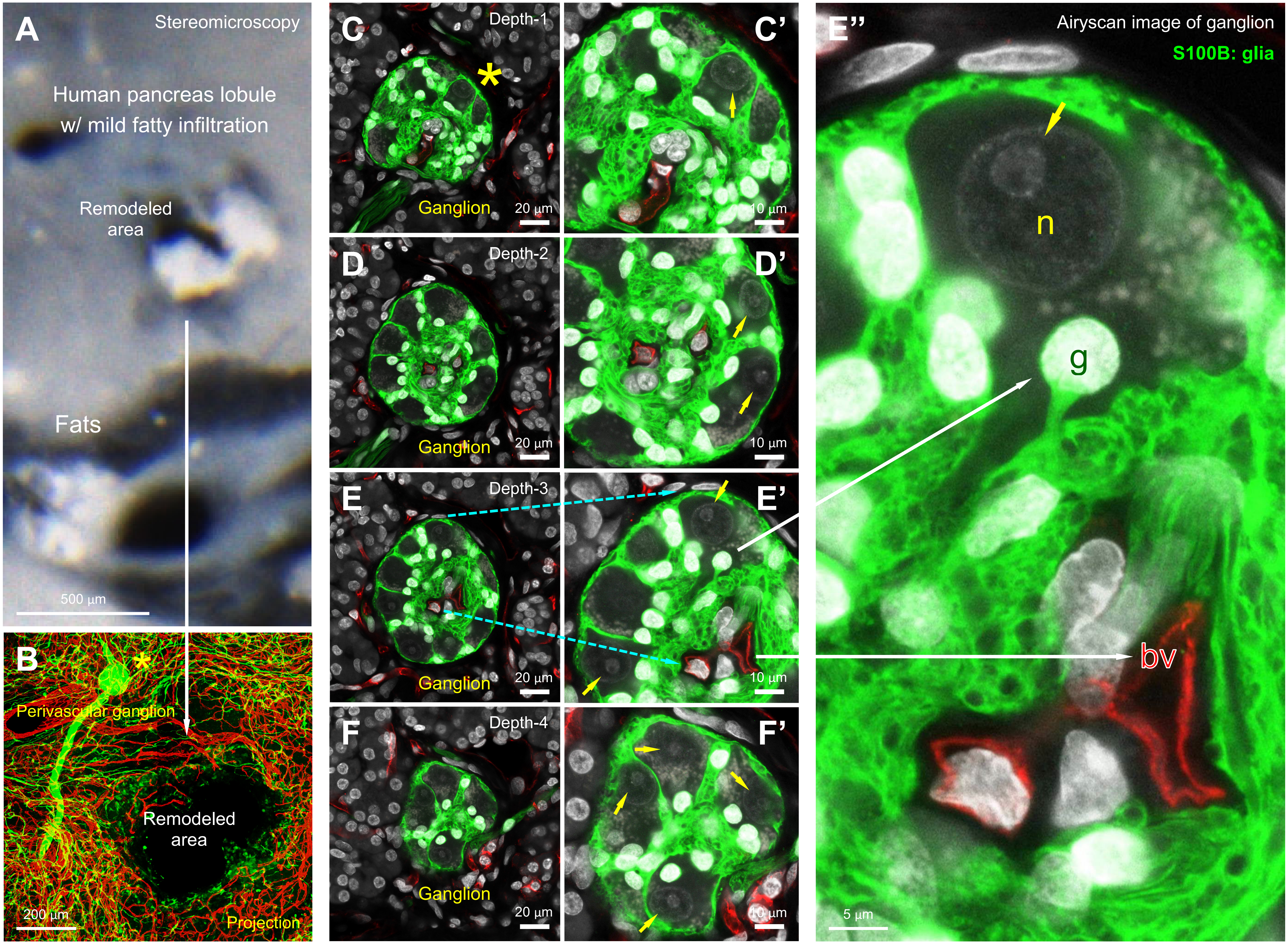

Supplementary Fig. 1 (related to Fig. 2)**. Human pancreas with mild steatosis: panoramic-to-Airyscan super-resolution neurohistology (S100B labeling of ganglion).**

(A) Pancreatic lobule affected by fat infiltration (male, 55 years old, body segment of donor pancreas). An arrow from panel **A** to **B** indicates a remodeled intra-lobular area.

(B) Projection of neurovascular networks in the remodeled area highlighted in panel **A** (arrow). Green: glial marker S100B; red: endothelial marker CD31. S100B staining detects a ganglion in the microenvironment (asterisk), which is further magnified in panels **C–F**.

(C–F) In-depth Airyscan imaging of the glial-neuronal association within the ganglion. Depth-resolved images identify neurons in the ganglion (panels **C–F**) with magnified views shown in panels **C'–F'**. Cyan arrows from **E** to **E'** illustrate the magnification. Yellow arrows in **C'–F'** indicate neurons, which display dimly stained nuclei with a distinct nucleolus and are surrounded by condensed S100B^{+} glial processes. In panel **E'**, the neuron, glia, and blood vessels are further magnified in **E''** (white arrows).

(E'') Magnified view of the ganglion. The neuron displays a visible nucleus and nucleolus, marked by a yellow arrow and labeled "n." Nearby blood vessels ("bv") are separated from direct contact with the neuron by condensed glial cell bodies ("g") and their intervening processes.
