# Supplementary material for: Human liver and pancreas innervation: resolving 3D neurohistological challenges and advancing insights
Source: J Biomed Sci. 2025 Nov 10;32:97. doi: 10.1186/s12929-025-01194-y (PMC12599030; doi:10.1186/s12929-025-01194-y)
Supplement: Supplementary file 5 — Additional file 5. [file 12929_2025_1194_MOESM5_ESM.docx]

**Supplementary Materials**

**Supplementary Video 1. Panoramic-to-3D Airyscan super-resolution neurohistology of human liver with steatosis.**

Left (still images): A liver lobule showing steatosis (ballooning hepatocytes) and sympathetic innervation. Green: tyrosine hydroxylase labeling of sympathetic nerves; magenta: CK19 labeling of bile ducts (indicating portal fields); white: DAPI labeling of nuclei and liver autofluorescence. The yellow asterisk and arrow indicate the area magnified in the movie. The lobules were obtained from the distal resection margin of hepatocellular carcinoma (male, 55 years old, right lobe).

Right (movie): Depth-resolved Airyscan imaging captures intra-lobular innervation in the steatotic liver. A 3D projection of the intra-lobular neural network is presented at 01:18–01:34. The video reveals sympathetic nerve fibers and varicosities at micron-to-submicron resolution, confirming direct sympathetic innervation of hepatocytes, including ballooning cells.

=====================================================================

**Supplementary Video 2. 3D Airyscan super-resolution neurohistology of human intrapancreatic ganglion: cholinergic neuron and VAChT⁺ vesicles on Nissl bodies.**

**Left (still images):** Lobular environment of human intrapancreatic ganglia. Magenta: PGP9.5 labeling of nerves and islets; yellow: VAChT labeling of parasympathetic nerves; white: DAPI labeling of nuclei. The yellow arrow and asterisk indicate the ganglion magnified in the inset and movie. The lobules were obtained from the body segment of a donor pancreas (male, 49 years old), immunolabeled and embedded in the high-refractive-index polymer (Fig. 5) for panoramic-to-Airyscan imaging (right).

R**ight (movie):** In-depth Airyscan imaging of VAChT⁺ vesicles on Nissl bodies (cyan). This recording demonstrates that the resolving power of 3D human pancreatic neurohistology is at the vesicle level.

=====================================================================

**Supplementary Video 3. 3D Airyscan super-resolution neurohistology of human intrapancreatic ganglion: the glial–neuronal association.**

Left (still images): Lobular environment of human intrapancreatic ganglia and surrounding islets. Magenta: PGP9.5 labeling of nerves and islets; green: S100B labeling of glia; white: DAPI labeling of nuclei. The projection of PGP9.5 and S100B signals (inset) highlights ganglionic–islet associations via abundant peri- and intra-lobular nerve extensions. Lobules were obtained from the tail segment of a donor pancreas (female, 51 years old), immunolabeled and embedded in high-refractive-index polymer (Fig. 5) for panoramic-to-Airyscan imaging. The asterisk indicates the ganglion magnified in the movie (right).

Right (movie): In-depth Airyscan imaging of the glial–neuronal association. Part 1 (00:00–00:43) shows signal projection (PGP9.5 and S100B) and in-depth recording of the intrapancreatic ganglion (left region, two neurons inside). Part 2 (00:44–01:46) highlights a third neuron on the right side of the ganglion (cyan asterisk). The video demonstrates how paired immunolabeling resolves closely associated neural structures—glial cell bodies and processes surrounding neurons—enabling cellular and subcellular characterization in 3D.

**Supplementary Videos 2 and 3** exemplify the depth-resolved ganglionic mapping discussed in the main text, highlighting how 3D imaging and polymer embedding enable panoramic, cellular, and subcellular insights essential for translational neuroendocrine research.

=====================================================================
